# Imaging Findings at the Quadrangular Joint in Carpal Boss

**DOI:** 10.5334/jbr-btr.1257

**Published:** 2017-04-25

**Authors:** Marc Mespreuve, Karl Waked, Koenraad Verstraete

**Affiliations:** 1 Department of Radiology, St.-Maarten General Hospital, Leopoldstraat 2, 2800 Mechelen, BE; 2 Department of Radiology, Ghent University, De Pintelaan 185, 9000 Ghent, BE; 3 Department of Surgery, Ghent University, De Pintelaan 185, 9000 Ghent, BE

**Keywords:** wrist, carpal boss, os styloideum, radiography, CT, US, MRI

## Abstract

A carpal boss was initially described as a bony, sometimes painful mass at the quadrangular joint. Clinical examination and plain radiography will usually reveal the diagnosis. US and CT may add information. MRI may illustrate a variable bony morphology and additional bony and soft tissue pathologies. Bone marrow edema shows a significant correlation with a painful carpal boss. Hence, MRI may be of additional diagnostic value in patients with persistent pain and preoperatively. This paper presents a review of the anatomy and pathology in carpal boss. The merit of each imaging modality – in particular MRI – is mentioned.

## Introduction

A carpal boss (CB) – as initially described by Fiolle [1] in 1931 – is a bony prominence at the dorsal side of the second or third carpometacarpal (CMC) joint. CB is often seen on imaging both in symptomatic and asymptomatic patients. Patients typically present with a local swelling and/or pain at the second or third CMC joint. Radiography and in particular Computed Tomography allows for evaluation of the bony morphology, but fail to correlate with the experienced pain. The presence of bony abnormalities alone does not allow to confirm or differentiate symptomatic findings in a large number of cases; neither does the absence allow exclusion of CB syndrome. Both techniques fail to illustrate the vast majority of the soft tissues lesions around the quadrangular joint (soft tissue edema, tendinopathy, ligamentous lesions, synovitis). Ultrasound and color Doppler may add information about tendinopathy and synovial proliferation in a number of cases. Magnetic Resonance Imaging may illustrate in detail an apparently variable bony morphology and also a variety of additional bony and soft tissue pathologies causing CB. Bone marrow edema (BME) around the quadrangular joint shows a highly significant correlation with a painful CB [2].

CB is usually treated conservatively (activity modification, particularly anti-inflammatory analgesics and eventually a wrist splint). If the pain persists, an injection of a long acting steroid is given additionally. In 70% to 80% of the patients the pain will remit over time. In patients with persevering pain, surgery may be considered [3,4]. The bony abnormality, the arthritic part of the joint and the soft tissue swelling are excised. The exact location and morphology of the CB may guide the surgeon, allowing more limited resection or shaving in order to prevent post-operative instability due to laceration of the small dorsal ligaments.

The aim of this pictorial review is to present an overview of the spectrum of pathology at the quadrangular joint (not to be confused with the quadrangular space or foramen humerotricipitale, a space located in the posterior compartment of the axillary region) in CB and to highlight the role of the different imaging modalities.

## Anatomy and Biomechanics

**Figure 1:**
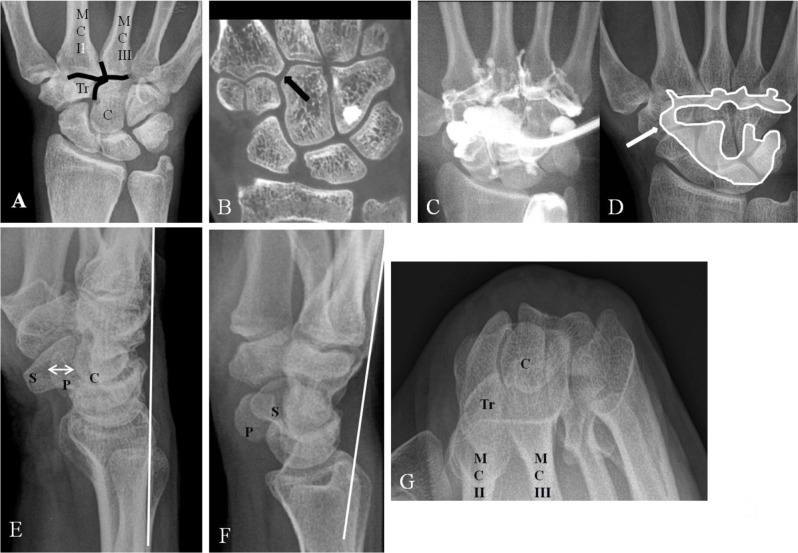
Normal anatomy of the quadrangular, midcarpal and (second to fifth) CMC joint. Plain radiography PA **(A)**; Cone beam CT coronal view **(B)**; Midcarpal arthrography **(C)** with schematic drawing **(D)**; and Plain radiography Lateral view **(E)**, Cuono view **(F)**, and Carpal bridge view **(G)**. **(A)** The quadrangular joint (black lines) between the base of the second and third metacarpals, the capitate bone and the trapezoid bone with. **(B)** detail of the quadrangular joint (arrow). **(C–D)** Normal communication between the midcarpal joint and the CMC joint (D; arrow). **(E)** The palmar border of the pisiform bone is in the middle between the palmar border of the scaphoid bone and the capitate bone (arrow). A tangent line is drawn parallel to the dorsum of the third MC for the evaluation of a bony prominence at the CMC joint (CB). **(F)** The pisiform bone is more prominent due to the supination. Tangent line parallel to the better exposed dorsum of the third MC. No bony prominence beyond the reference line. **(G)** Carpal bridge view of the quadrangular joint (MC II = second metacarpal, MC III = third metacarpal, C = capitate bone, P = pisiform bone, S = scafoid bone, Tr = trapezoid bone).

**Figure 2:**
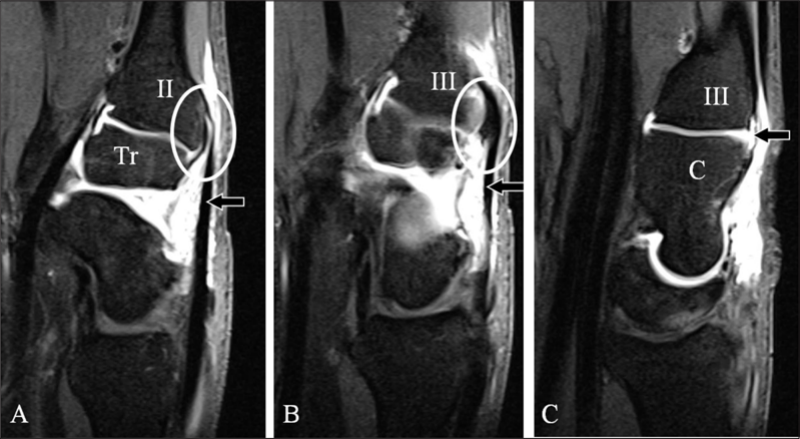
Normal anatomy of the dorsal side of the second CMC and third CMC joint. **(A–C)** MR midcarpal arthrography sagittal SE T1-WI FS. **(A)** Trapezoid (Tr) – second metacarpal (II) joint showing the close relationship (oval) of the dorsal CMC ligament and the tendon of the ECRL (arrow). **(B–C)** Third CMC joint showing the close relationship (B; white oval) between the tendon of the ECRB (B; arrow) and the dorsal CMC ligament (C; arrow) between the third metacarpal and the capitate bone **(C)**.

A CB appears almost exclusively in the quadrangular joint of the wrist (Figure 1A–B) at the second and third CMC joint space [5], although in approximately 39% other locations are possible [2]. The shape of the base of the second metacarpal (MC) fits into the trapezoid bone as an inverted *V*, as does the third MC base into the capitate bone (Figure 1). In this small region, the MC bases, the dorsal CMC ligaments and the insertion of the extensor carpi radialis longus (ECRL) and brevis (ECRB) tendon (respectively at the base of the second and third MC) are all in very close relationship to each other (Figure 2). The complex M-shaped CMC (second to fifth) joint is in connection with the midcarpal joint (Figure 1C–D) through the joint space between the trapezium and trapezoid bone. It is highly constrained by its joint contours and the ligamentous anatomy around the quadrangular joint [2,6]. The range of motion at the CMC joint is less than 5° [7].

## Etiology

The etiology of CB remains unclear. Various etiologies (carpal coalition, traumatic or degenerative) have been described: a congenital predisposition, the presence of an os styloideum (OS), a childhood fracture, microtraumata with a rupture of a dorsal ligament, a traumatic periostitis secondary to chronic traction on an extensor carpi radialis tendon insertion or an overgrowth of bone in response to abnormal stress, an osteophyte, or an exostosis [3,9].

## Demography

CB usually presents in the fourth decade of life. However, some authors noticed earlier cases during the second and third decade [10] and sometimes even in adolescents [2,11]. The literature does not describe gender predominance. CB is present in 8–26% of the general population. It is a bilateral finding in up to 21% of the patients [12]. Symptomatic CB is mostly found in the dominant hand-wrist [13,14], although other series describe almost no difference between the right and left side [2].

## Clinical Manifestation

Most protuberances at the CMC joint are asymptomatic ganglion cysts with only cosmetic complaints. Hence a bony CB is clinically often misinterpreted as a ganglion cyst. They are usually located more proximally in relation to the CMC joint and often have a soft consistency [8].

Symptomatic patients with swelling and/or pain at the second or third CMC joint most likely have a bony protuberance [4]. The patients experience pain at the dorsal side of the CMC joint at the end of flexion and extension. Resisted extension or hyperextension aggravates the pain. Chronic pain may be related to secondary degenerative osteoarthritis, an inflamed ganglion cyst or extensor tendon subluxation [15]. Repetitive strain may aggravate the symptoms due to a tenosynovitis of the ECRL or ECRB tendon. CB of either etiology limits the lateral movements of these extensor tendons [16], or causes subluxation, contributing to the symptomatology. The presence of an OS alone does not allow for the diagnosis of symptomatic CB as the incidence in the general population with CB is up to 8–26%, with less than 3% being symptomatic [10].

## Imaging Techniques

### Radiography

The basic radiological views of the wrist are a PA and a tangential view. There are two different radiological tangential projections for the diagnosis of CB: the Cuono view (or Carpal Boss view) (modified lateral view with the hand supinated (30–40°) and in a 20–30° ulnar deviation) (Figure 1F) and the carpal bridge view (90° palmar flexion of the wrist, dorsal side of the hand on the film and a 45° beam angulation in superoinferior direction towards the wrist) (Figure 1G). The Cuono view is considered the most practical and reliable [4,13] to demonstrate the bony prominence. However, bony superposition from the surrounding carpal bones may impede the evaluation of the exact morphology and even the detection of an OS or a fracture. Soft tissue evaluation remains very restricted (calcifications or indirect signs such as subcutaneous fat line obliteration and soft tissue swelling). The presence of bony anomalies illustrated by plain radiography alone does not allow to confirm CB in case of symptomatic findings in a large number of cases, neither does the absence of bony anomalies on radiography allows to exclude a CB syndrome [10].

#### Ultrasound

High frequency transmitters (15–18 mHz) allow for clear visualization of the tendon insertion of the ERCL and ECRB and the external outline of some of the dorsal ligaments. Fluid containing structures such as ganglion cysts will be illustrated. color Doppler may add information about active inflammatory synovial proliferations. However, bony structures and the associated pathology are hardly noticed.

#### Computed Tomography

With its lower radiation dose and higher resolution, multiplanar cone beam CT (Figure 1C) should be the first choice for the three-dimensional imaging of the bony anatomy and pathology of the quadrangular joint. Because of the elimination of superposition, the exact anatomy of the quadrangular joint will be documented in detail and small bony lesions such as avulsion fractures at the insertion of the overlying tendons may be revealed. Soft tissue lesions around the quadrangular joint are difficult to illustrate.

#### Magnetic Resonance Imaging

**Figure 3:**
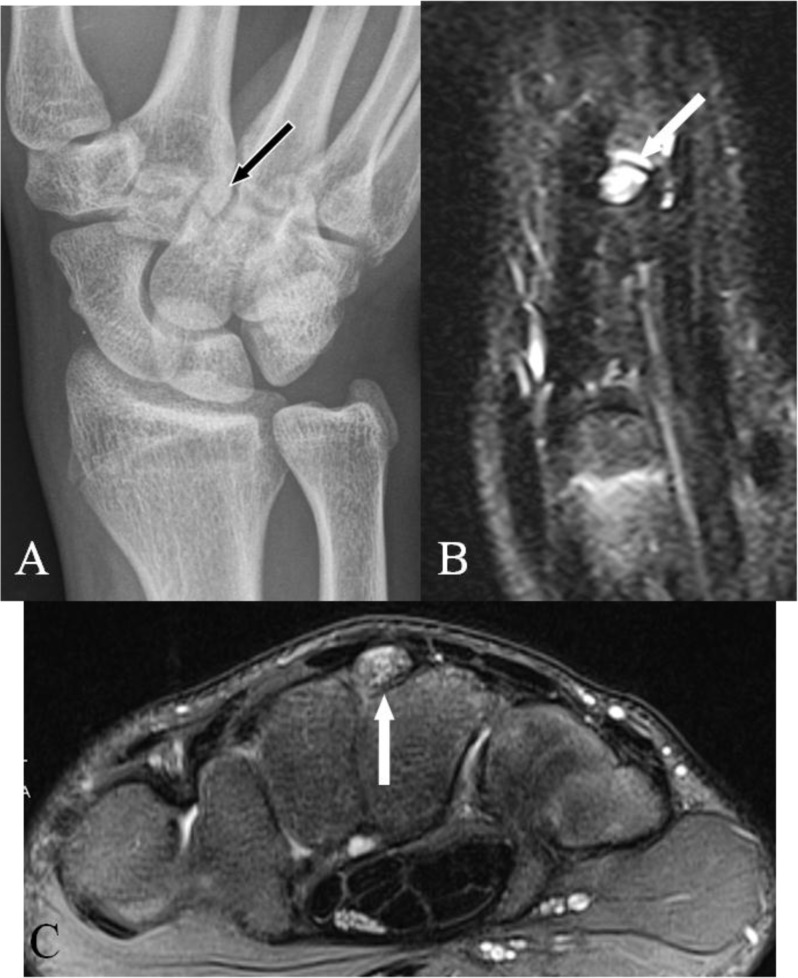
Acute painful CB in a tennis player. **(A)** Plain radiography PA view, **(B)** Coronal SE T2-WI FS and **(C)** Axial SE PD-WI FS. **(A)** Presence of an os styloideum (arrow). **(B–C)** Extensive bone marrow edema of the os styloideum (arrows), extending to the base of the third MC **(B)** (arrow).

All MRI examinations should be performed using a dedicated wrist coil. Isotropic 3D-GRE sequences are most suited for the anatomic evaluation of the bony margins of the quadrangular joint. MRI detects a CB in approximately 25% more patients compared to radiography [2]. In about 15% an OS which is not visible on radiography may be found [2]. The presence and exact location of BME is only revealed by fluid sensitive sequences (FS-T2-WI or STIR sequences), noticed as a high signal area around (a part of) the quadrangular joint (Figure 3B). Accessory bony coalitions will be illustrated and BME may reveal their clinical significance [17,18]. A fracture at the base of the metacarpal is usually clearly seen, also due to the absence of bony superposition (as for CT) and the accompanying BME (not visible on CT). Moreover, MRI adds a lot of information about the soft tissues without the use of radiation. A tear of the dorsal ligaments may be revealed. MRI after Gadolinium enhancement may be useful in case of inflammatory synovial proliferation. A midcarpal MR arthrography (Figure 2) may be needed to illustrate dorsal ligamentous lesions or a tendon rupture at the insertion of the ECRL or ECRB.

## Pathology

### Os Styloideum and morphology of Carpal Boss

The OS is an accessory [19] and immobile carpal bone between the trapezoid bone, the capitate bone and the base of the second and third MC [8]. It is believed to originate from a separate ossification centre (primordial carpal unit) [16], which normally fuses with the third MC to form the styloid process [8]. When the fusion does not occur, a separate ossicle – known as the OS – remains. This dorsal ossicle was already described by Saltzmann in 1725 [13,16]. The OS has an estimated prevalence of 1–4% [12,20] of the normal population. It may be isolated (2%) or more commonly fused to either the second or third MC (94%), the capitate bone (3.5%) or the trapezoid bone (0.5%) [13]. It has also been described as a ninth carpal bone [16], a metastyloid or parastyloid process [21]. This bony anomaly in the quadrangular joint – with or without partial osseous coalition – was even present in up to 63% in surgical studies on CB [3,5]. Since the dorsal ligamentous structures are absent in the presence of an OS [11], it is hypothesized that CB represents a congenital carpal coalition [5,7], which may cause symptoms in case of a fracture of the fibrous coalition similar to lunotriquetral fibrous coalition Minnaar type 1 [17]. The bilateral occurrence of CB is however less frequent (11–21%) [6] than in carpal coalition (in up to 61%). There is also a contrasting high prevalence of an OS in CB (33%) in relation to the low appearance in normal wrists (1–3%) [20]. Some authors even prefer the term CB syndrome referring to the association of an OS and wrist pain [22].

**Figure 4:**
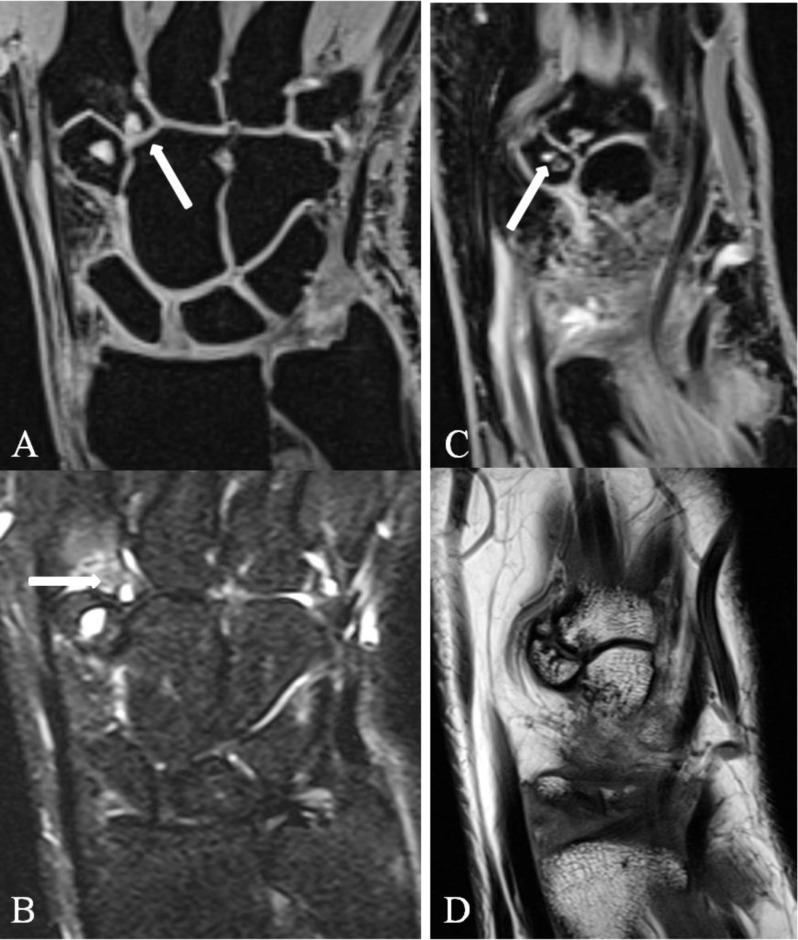
Dorsal anatomical variants. Coronal **(A)** 3D-GRE, **(B)** SE T2-WI FS, **(C)** 3D-GRE and **(D)** SE T1-WI. Small dorsal joint between **(A–B)** the second MC base and the os capitatum with edema and subchondral cysts at the base of the second MC (arrows) and **(C–D)** between the trapezoid bone and the third MC base with subchondral cysts on both sides of the joint (C; arrow).

On plain film and in particular on CT and MRI the presence of an OS and the precise location and exact bony morphology of the CB are illustrated (Figure 3). Due to bony superposition, radiography may often not reveal all of the CB and/or OS and in particular not the exact location and extent of the CB. The different possible quadrangular joint components should be analysed in detail: the bases of the second and third MC; the joint between the OS and the third MC base; the process of the third MC base; the joint between the OS, capitate bone, and third MC base; and also dorsal anatomical variants such as a small dorsal joint, usually located between the trapezoid bone and the third MC base, or less frequent between the second MC base and the os capitatum (Figure 4).

Presence and exact location of BME in CB is only revealed by MRI and is strongly correlated with the presence of clinical symptoms [2]. MRI analysis of joint morphology of CB in 57 patients [2] showed that in a majority of 28 patients the CB was located at the third CMC joint, in 12 patients at the joint between OS and the third MC, in 7 patients at the second CMC joint and in 10 patients at other parts of the quadrangular joint.

## Carpal Coalition

Although carpal coalition usually is a coincidental finding and mostly asymptomatic, it may cause pain and discomfort [17,18] due to alterations in the normal mechanics of the wrist. Carpal coalitions were only reported in the area of the second and third CMC joint in a cadaveric study of the second to fifth CMC joint [5,7]. In case of a rare trapezium-trapezoid or trapezoid-capitatum coalition in association with a CB, the loss of movement between the fused bones may result in a compensatory increase of movement and stress at the joints between the synostosis and the surrounding bones [23]. An extremely rare case of a CB arising from an accessory capitate bone located between the third and fourth metacarpals and the capitate and hamate bone has also been described [24]. The Minnaar classification (type 1 = fibrous; type 2 = partial osseous; type 3 = complete osseous; type 4 = type 3 with other carpal anomalies) [17] is used for the evaluation of a bony coalition in this region. A synchondrosis, eventually with surrounding increased signal intensity due to a reactive BME may be evaluated by MRI.

## Traumatic Bone Lesions

**Figure 5:**
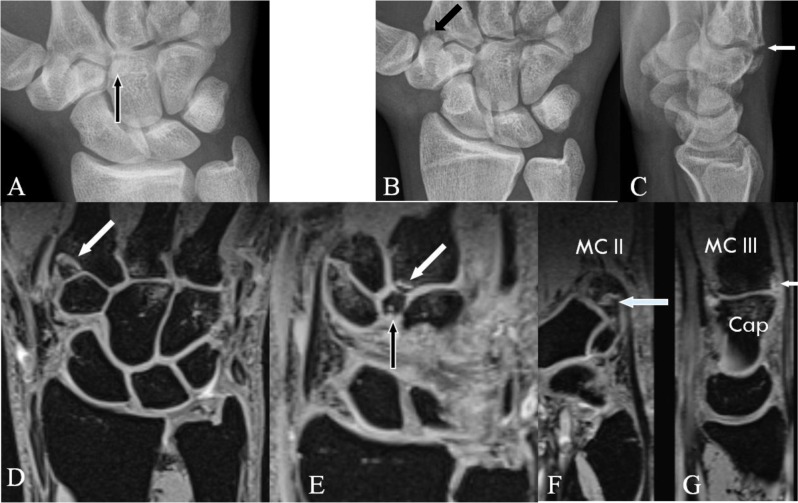
Recent fracture at the base of the second and third MC. **(A–C)** Plain radiography, **(D–E)** Coronal 3D-GRE and **(F–G)** Sagittal 3D-GRE. **(A)** Asymptomatic presence of an OS (arrow) at the CMC joint in a previous radiograph. **(B–G)** The patient presented three years later with elective pain at the CMC joint following trauma. **(B)** Fracture at the base of the second MC (arrow), **(C)** CB with an os styloideum (arrow), **(D, F)** fracture at the base of the second MC (arrow) and **(E, G)** the base of the third MC (only visible on MRI) (white arrow), **(E)** presence of an os styloideum (black arrow) and **(G)** rupture of the dorsal CMC ligament (arrow) with surrounding soft tissue edema. (MC = metacarpal; Cap = os capitatum).

A traumatic injury (23–27%) [3] after a dorsal wrist trauma at the quadrangular joint region may cause an acute CB (Figure 5): a fracture of the MC base, an avulsion fracture at the insertion of the ECRL or ECRB tendon, or even a direct lesion of the OS. CB may be caused by either a stress-induced hypertrophy of the bone or by the healing of micro-fractures resulting from abnormal high stress, in particular repetitive forced extension (hammering, golf, tennis) [3,25] applied on these very rigid joints. The major axes of stress on the wrist are known to run through the scapholunate and the quadrangular joint [26].

Acute BME after a direct injury of the OS was described in an ice hockey player [3] and in a tennis player (Figure 3) [27]. In particular, in athletes, hand and wrist injuries are frequent and account for 3% to 9% of all injuries. In collision sports, the incidence may rise up to 15% [28]. In golf and racquet sports it is even a rather common injury [14]. BME is noticed more often in younger patients, which suggests a (micro) traumatic aetiology rather than a degenerative one.

**Figure 6:**
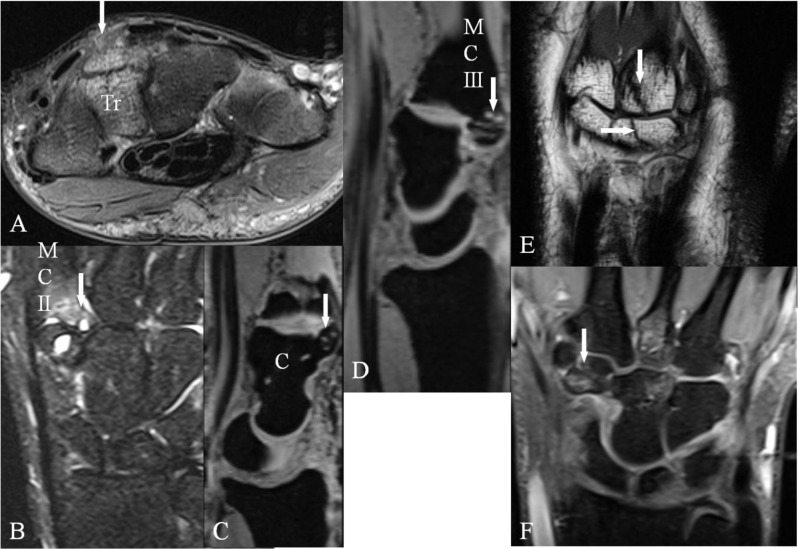
Old, complex traumatic lesions of the different components of the quadrangular joint at the origin of a CB. **(A)** Axial SE PD-WI FS, **(B)** Coronal SE T2-WI FS, **(C–D)** Sagittal 3D-GRE, **(E)** Coronal SE T1-WI and **(F)** Coronal SE PD-WI FS. **(A)** Fracture in the coronal plane of the os triquetrum (arrow). **(B)** Posttraumatic deformity of the base of the second MC with bone marrow edema (arrow). **(C)** Posttraumatic deformity of the dorsal side of the capitate bone (arrow). **(D)** Posttraumatic deformity of the base of the third metacarpal (arrow). **(E–F)** Multiple fractures of the base of the third MC **(E)** (vertical arrow), the capitate bone **(E)** (horizontal arrow) and of the trapezoid bone (F; arrow).

**Figure 7:**
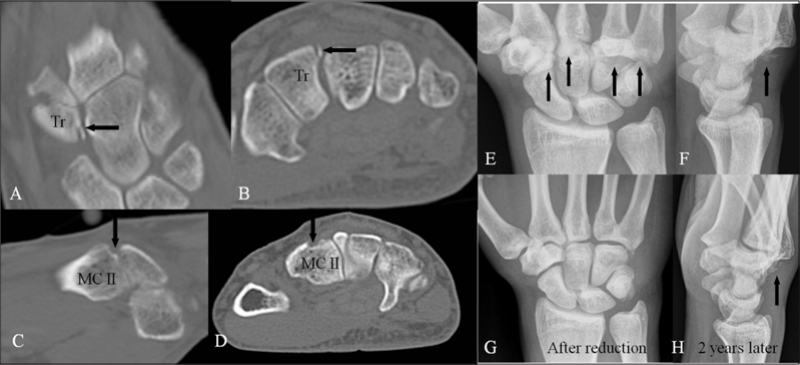
Traumatic lesions around the quadrangular joint. CT-scan **(A–D)** with coronal **(A)**, axial **(B, D)** and sagittal **(C)** reconstructions and plain radiography **(E–H)**. **(A–B)** Fractures with loose bony fragments of the trapezoid bone and **(C–D)** at the base of second MC, which may cause a posttraumatic CB. **(E–F)** Severe traumatic luxation at the CMC joint (arrows). **(G)** Normal M-form of the CMC joint after reduction. **(H)** Chronic subluxation at the CMC joint (arrow) two years later. Bony prominence simulating CB.

The bony CB prominence may also be caused by a non-united fracture. Old traumatic lesions, small or complex (Figure 6), may be shown. In old bony trauma, the initial CT (Figure 7A–D) may confirm the diagnosis of CB as callus formation may be seen on small chip fractures around the quadrangular joint. Chronic symptoms may be related to recurrent (sub)luxation (Figure 7E–H), joint degeneration, an inflamed ganglion cyst, a bursa or an extensor tendinopathy and chronic (hyperextension) stress (piano players).

Fractures at the quadrangular joint are often concealed on radiography because of the complexity of this joint. Small and complex fractures are best illustrated by cone beam CT. A diffuse signal increase in case of symptomatic BME of the OS (if present), the base of the second or third MC or the adjacent carpal bones is well depicted on fluid sensitive sequences, as is regional soft tissue edema in acute injuries. The absence of BME seems to have a relatively high negative predictive value (83%) and the presence of BME has a less positive predictive value (73%) [2].

## Degeneration

**Figure 8:**
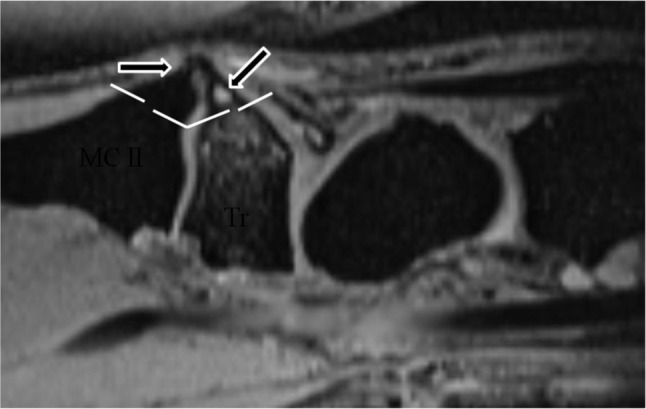
*Volcano*–*type* osteophytes. 3D-GRE sagittal view. Osteophytes (horizontal arrow) resembling a volcano, with subchondral cyst (oblique arrow) and irregular narrowing of the dorsal joint space. Location of a wedge resection (dotted line) of the CB on the dorsal articular surface.

An osteophyte, in case of secondary highly localized degenerative osteoarthritis, or an exostosis may cause CB. As the range of motion is less than 5° and CB is also diagnosed in younger patients [5], it seems very likely that these osteophytes are rather a result of secondary degenerative osteoarthritis. Degeneration at the quadrangular joint presents with the typical bony overgrowth on the dorsal side of the CMC joint, described as *volcano-type* osteophytes (Figure 8) or *beaking/bec de perroquet* – osteophytes [9,10]. Degenerative osteophytes are a frequent finding. There may be an accompanying joint effusion in the CMC joint or thinning of the cartilage layer. An associated intra-osseous ganglion may be found.

**Figure 9:**
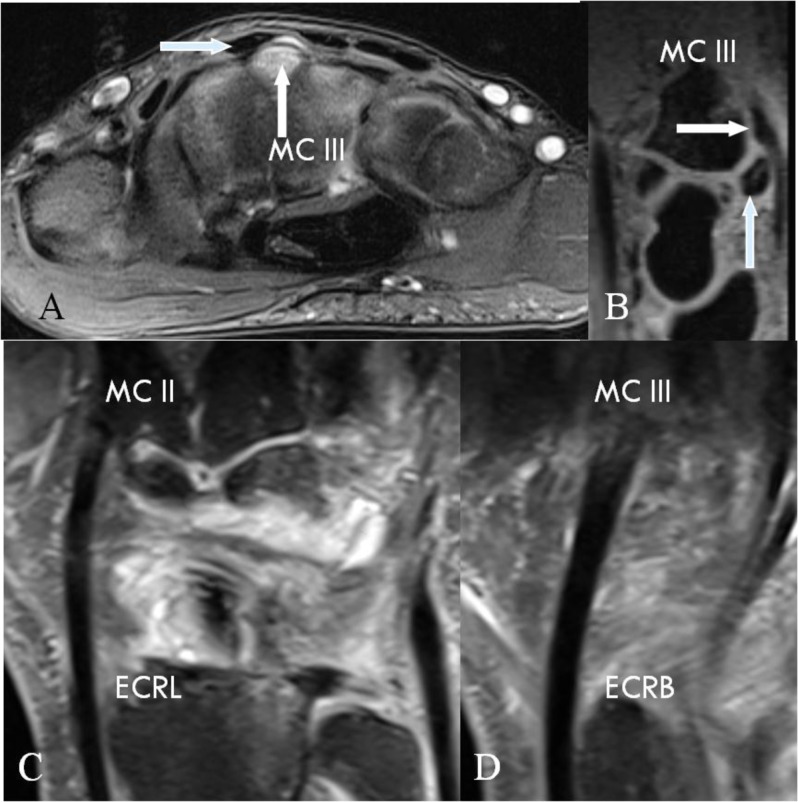
Interference of CB with the course of the ECRL or ECRB. **(A)** Axial SE PD-WI FS, **(B)** Sagittal 3D-GRE, **(C–D)** Coronal SE PD-WI FS. **(A–B)** CB due to an os styloideum (vertical arrows) containing BME. Interference with the normal path of the ECRB tendon (horizontal arrows). **(C)** Path of the ECRL to the radial side of the second MC and **(D)** the ECRB to the radiodorsal side of third MC. Swelling, intra- and peritendinous high signal at the insertion of the ECRB tendon on the third MC.

Dorsal osteophytes (even small ones), the exact location, the bony morphology and small subchondral cysts are clearly visualized on CT and 3D-GRE isotropic sequences in the sagittal plane. Tendinitis or tendinosis and BME – due to the mechanic interference of the CB with the normal path of the ECRL or ECRB (Figure 9) – are well illustrated on MRI.

## Ganglioncysts

**Figure 10:**
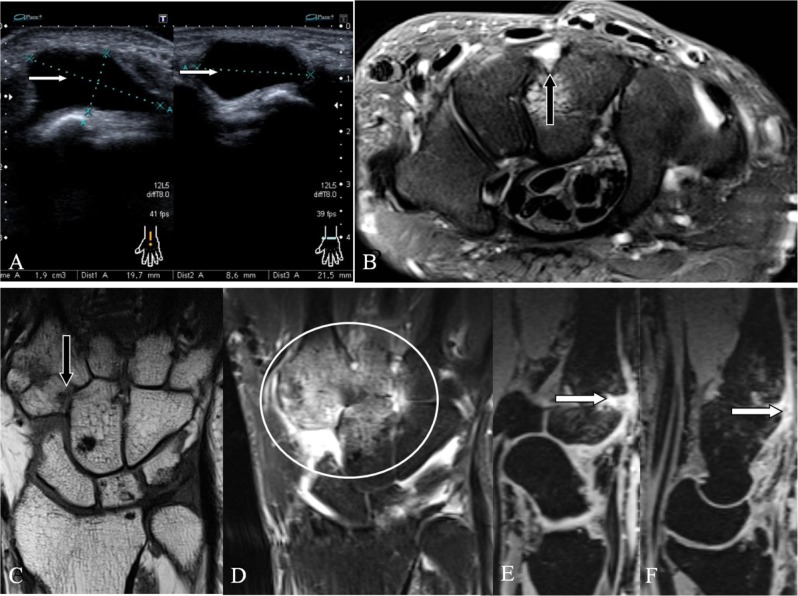
CB due to a soft tissue lesion. **(A–B)** A dorsal ganglion cyst (arrows) in two different patients. **(A)** Ultrasound, **(B)** Axial SE PD-WI FS. Protuberance at the CMC joint due to a large **(A)** and a small **(B)** dorsal ganglioncyst (arrows), both asymptomatic. **(C–F)** Contrast enhancement in CB due to rheumatoid arthritis. **(C)** Coronal SE T1-WI, **(D)** Coronal SE T1-WI FS with gadolinium contrast, **(E–F)** Sagittal 3D-GRE. **(C)** Arthritis with erosion at the quadrangular joint (arrow). **(D)** Prominent contrast enhancement around the quadrangular joint (oval). **(E–F)** Dorsal prominent synovitis (arrows) at the **(E)** second and **(F)** third CMC joint.

CB patients may present with a cyst or de novo bursitis at the dorsal side of the quadrangular joint. These protuberances are usually asymptomatic with only cosmetic complaints. A ganglion cyst often appears as a soft tissue mass without calcifications [10]. In most cases, US (Figure 10A) will reveal the exact diagnosis. However, MRI is of additional value to detect small ganglion cysts (Figure 10B) as seen in up to 30% [10].

## Rupture of a Dorsal Ligament at the CMC Joint

An acute CB in an elite swimmer was related to a tear of the dorsal ligament between the capitate and the OS [29]. A tear of the dorsal ligaments between the base of a MC and the adjacent carpal bone may be revealed on MRI in recent trauma (Figure 5G). However, additional MR midcarpal arthrography may be required in equivocal cases to illustrate these subtle lesions.

## Tendon Rupture

A disruption of the distal tendon attachment of the ECRL or ECRB tendon may be assessed and/or an avulsion fracture at the insertion of these same tendons may be revealed by MRI, although small fracture fragments may be missed and additional cone beam CT may be necessary.

## Inflammatory Pathology

Clinical diagnosis of an isolated dorsal soft tissue mass may be related to an (isolated) active arthritis (Figure 10C–F) with synovitis (Figure 10E–F), well-illustrated by contrast enhancement on MRI (Figure 10D). There may be an accompanying joint effusion in the CMC joint, erosions (Figure 10C) or thinning of the cartilage layer. The superficial location allows US to illustrate the presence of this small soft tissue lesion as well. Color Doppler may illustrate the hypervascularisation.

## Therapy

CB is usually treated conservatively with non-steroidal anti-inflammatory drugs and the pain will often diminish over time.

The detection of (small) fractures, BME or osteophytes and the knowledge about the presence of tendinous or ligamentous pathology may allow more accurate conservative therapy planning. When surgery (**Figure 8**) is considered, the exact location and bony morphology of the CB may allow a more limited resection or shaving in order to preserve the small ligaments around the quadrangular joint. Sometimes, they are damaged during surgery which may cause instability.

After conservative therapy or surgery, MRI may be useful for the follow-up as for evaluating the regression of BME. The (complete) resection of osteophytes and associated ganglion cysts and the reinsertion of the extensor tendon(s) may also be evaluated.

## Conclusion

CB at the quadrangular joint is relatively unknown, but frequent in daily practice. Clinical examination and plain radiography may reveal the diagnosis in most cases and will lead to conservative treatment. Certainly in case of persistent pain and in the preoperative setting, more extensive imaging should be considered as different pathologies may be at the origin of the CB. US adds information about the presence of an (occult) ganglion cyst, dorsal tendon pathology and (possibly) synovitis. MRI (without the use of radiation) and (cone beam) CT may detect occult fractures and allow for a better evaluation of the variable bony morphology of CB. A variety of soft tissue pathologies causing CB may additionally be illustrated by MRI. BME around the quadrangular joint – which is only visible on fluid sensitive MRI sequences – highly correlates with a painful CB.

## Important Teaching Points

A carpal boss may be more than a bony prominence and a variety of locations around the quadrangular joint are possible.

In case of persistent pain and in the preoperative setting, more extensive imaging should be considered.

A variety of soft tissue pathologies causing carpal boss may additionally be illustrated by MRI.

BME around the quadrangular joint – illustrated only by MRI – highly correlates with a painful carpal boss.
